# Tissue Doppler Imaging, Pulmonary Haemodynamics and Conventional Echocardiographic Indices for Early Bronchopulmonary Dysplasia Prediction in Preterm Infants: A Prospective Observational Feasibility Study

**DOI:** 10.3390/children13050660

**Published:** 2026-05-08

**Authors:** Wisam Muhsen, Joanne Hosking, Jos M. Latour, Eirik Nestaas

**Affiliations:** 1 Faculty of Health, University of Plymouth, Plymouth PL6 8BU, UKnestaas@hotmail.com (E.N.); 2 University Hospital Plymouth, Derriford Road, Plymouth PL6 8DH, UK; 3 Medical Statistics, Faculty of Health, University of Plymouth, Plymouth PL6 8BU, UK; 4 Department of Nursing, Zhongshan Hospital, Fudan University, Shanghai 200032, China; 5 Curtin School of Nursing, Curtin University, Perth, WA 6102, Australia; 6 Institute of Clinical Medicine, University of Oslo, Postboks 1171 Blindern, 0318 Oslo, Norway; 7 Clinic of Pediatrics and Adolesence, Akershus University Hospital, Sykehusveien 25, Nordbyhagen, 1478 Lørenskog, Norway

**Keywords:** broncho-pulmonary dysplasia, echocardiography, normalisation to cardiac size, prediction model development, preterm infants, tissue Doppler Imaging

## Abstract

**Highlights:**

**What are the main findings?**

- Right ventricle (RV) function and pulmonary vascular changes detectable by serial echocardiography in the first 10 days of life can distinguish preterm infants with evolving broncho-pulmonary dysplasia (BPD) from unaffected infants.
- When echocardiographic measurements are normalised to cardiac size, a compensatory hyperdynamic right ventricular response related to preterm infants with evolving BPD can be revealed; notably, all preterm infants with reversed anterior cerebral artery diastolic flow subsequently developed BPD.

**What are the implications of the main findings?**

- The feasibility of integrating routine clinical risk factors with systematic echocardiographic assessment of RV function and pulmonary haemodynamics provides a compelling multi-domain foundation for developing a BPD prediction model that extends beyond conventional demographic variables.
- This approach holds the potential to identify high-risk preterm infants early enough to intervene during a critical therapeutic window, and to enhance the efficiency of future trials through targeted enrolment of those most likely to benefit.

**Abstract:**

**Background/Objectives**: Bronchopulmonary dysplasia (BPD) remains a major complication of prematurity, yet no prediction model incorporating echocardiographic assessment of right ventricle (RV) function and pulmonary haemodynamics exists. This study aimed to identify clinical and echocardiographic candidate variables of BPD to inform the design of a future multicentre study for BPD prediction model construction. **Methods**: This prospective observational feasibility study recruited preterm infants born before 32 weeks of gestation. Echocardiographic scans were performed at Day 5 and Day 9 postnatally. Candidate variables across six domains, clinical, RV systolic, diastolic, and global function, pulmonary-haemodynamics, and patent ductus arteriosus (PDA) variables, were evaluated using Mann–Whitney U tests and univariable logistic regression. **Results**: Of 40 preterm infants enrolled, 27 (68%) developed BPD. The BPD group had lower gestational age (median 26 vs. 30 weeks, OR 0.50, *p* < 0.001), lower current weight (median 763 vs. 1200 g, OR 0.54, *p* = 0.002), and higher mean airway pressure (OR 1.86, *p* = 0.002). By Day 5, significant differences included higher normalised RV s’ (OR 2.26, *p* = 0.042), shorter RVET (OR 0.49, *p* = 0.018), elevated RV-MPI-PW (OR 2.28, *p* = 0.019), shorter PAAT (OR 0.72, *p* = 0.047), and larger normalised PDA diameter (OR 1.70, *p* = 0.002). By Day 9, normalised RV a’ (OR 1.93, *p* = 0.015), RV E/e’ ratio (OR 2.03, *p* = 0.033), and RV e’/a’ ratio (OR 0.54, *p* = 0.019) additionally emerged. All infants with reversed anterior–cerebral–artery diastolic flow subsequently developed BPD. **Conclusions**: Clinical and echocardiographic variables across multiple RV-function, PDA and pulmonary vascular domains were identified as candidate variables for BPD, providing a conceptual framework for a future multicentre prediction model study.

## 1. Introduction

Bronchopulmonary dysplasia (BPD) remains the most prevalent chronic respiratory morbidity affecting preterm infants, representing a significant clinical challenge in contemporary neonatal medicine [1,2]. First described by Northway and colleagues in 1967, the clinical phenotype of BPD has evolved substantially over the past five decades, transitioning from a disease characterised by airway injury and fibrosis to the ‘new BPD’ of the post-surfactant era, which is predominantly defined by arrested alveolar and pulmonary vascular development [3,4]. Despite advances in antenatal corticosteroids, surfactant therapy, and respiratory support, BPD incidence has not declined and may be rising in some populations [5,6]. Improved survival of extremely preterm infants has been accompanied by a rise in BPD incidence, as increasingly vulnerable neonates now survive [7].

The clinical significance of BPD extends far beyond the neonatal period, as affected preterm infants experience substantial short-term and long-term sequelae [8,9]. Pulmonary hypertension (PH) represents one of the most consequential cardiovascular complications associated with BPD [10,11]. The presence of BPD-associated PH confers a substantially elevated mortality risk compared to infants with BPD alone [12]. Beyond cardiovascular complications, BPD is associated with adverse neurodevelopmental outcomes, including, for example, delays in fine and gross motor skills and in cognitive function [13,14].

Given the morbidity and mortality burden associated with BPD, considerable research effort has been directed towards the development of clinically viable prediction models capable of identifying high-risk infants early in the disease trajectory, thereby facilitating targeted preventive interventions and optimised resource allocation [15]. Traditional risk prediction approaches have primarily employed logistic regression (LR) methodologies utilising maternal and neonatal clinical indices, with gestational age and birth weight consistently emerging as the variables with high predictive value [16,17]. Recent investigations have increasingly adopted nomogram-based prediction models, which offer intuitive visual representations of individualised risk estimation. Gao et al. (2023) developed a nomogram incorporating maternal age, delivery mode, birth weight, gestational age, invasive ventilation requirement, and haemoglobin levels; however, this model is still to be externally validated [18]. Their multivariable-based nomogram achieved an AUC of 0.905 with adequate calibration in the validation cohort, though the study was limited by its single-centre design and relatively small sample size (n = 237) [18]. Similarly, Sucasas-Alonso et al. (2024) [19] demonstrated the utility of competing risk methodology in constructing early prediction nomograms. Their day one model, incorporating birth weight, days since rupture of membranes, and surfactant requirement, yielded an AUC of 0.896 (95% CI, 0.792–0.999), while the day three model substituted fraction of inspired oxygen (FiO_2_) for membrane rupture duration, with a comparable AUC of 0.891 (n = 306). However, the models relied on conventional clinical variables, and echocardiographic data were limited to patent ductus arteriosus (PDA) assessment without incorporation of the right ventricle (RV) functional or pulmonary haemodynamic variables. The attenuation of FiO_2_ as a predictor beyond postnatal day three may reflect the confounding influence of transitional circulation, potentially limiting the reliability of risk estimation during these early postnatal days [19].

Furthermore, recent systematic reviews show that most existing tools rely on static clinical and demographic variables rather than dynamic physiological biomarkers that may better reflect evolving cardiopulmonary pathophysiology [20,21]. Romijn et al. (2023) [20] reviewed 65 studies containing 158 developed and 108 validated models. Although the median c-statistic results were satisfactory (0.84 for the developed and 0.77 for the validated models), there were concerns regarding the risk of bias [20]. Similarly, Kwok et al. (2023) [21] identified 53 prediction models across 64 studies; risk of bias was identified in 97% of studies, and 61% were single-centre studies. Both reviews emphasised the paucity of external validation [20,21]. Furthermore, where echocardiographic variables were included, these were largely limited to PDA assessment, omitting RV function and pulmonary haemodynamics that reflect the broader haemodynamic changes in evolving BPD.

Echocardiographic assessment of RV function and pulmonary haemodynamics offers a potentially valuable, non-invasive approach to identifying early markers of pulmonary vascular disease and impaired cardiac adaptation in at-risk preterm infants [22,23]. The BPD pathogenesis involves arrested pulmonary vascular and alveolar development, and echocardiographic variables of pulmonary vascular disease are detectable within the first postnatal week and predictive of subsequent BPD [1,24]. Integrating such echocardiographic variables with established clinical prediction therefore holds promise for improving prediction model performance and enabling earlier, targeted interventions. Approximately 25% of infants with moderate-to-severe BPD develop PH, characterised by abnormal vascular remodelling and increased pulmonary vascular resistance (PVR) [25]. The PDA represents a potentially modifiable risk factor, as a persistent left-to-right shunt increases pulmonary fluid filtration and impairs pulmonary dynamics [26]. Recent evidence demonstrated an association between the extended exposure to PDA and pulmonary vascular disease, in particular the development of BPD-associated PH [27,28]. These complex interactions support including pulmonary haemodynamics, PDA, and RV function as variables in a BPD prediction model.

The present feasibility study, designated REPORT-BPD (exploring Right vEntricular function applicability in a Prediction mOdel to identify pReterm infanTs with early Broncho-Pulmonary Dysplasia), aims to evaluate the potential utility of serial echocardiographic assessment in predicting BPD development among preterm infants born at less than 32 weeks’ gestational age. Specifically, we sought to: (1) Characterise the echocardiographic profiles of the participating preterm infants who subsequently develop BPD compared to those who do not. (2) Assess candidate echocardiographic variables to include in the BPD prediction model construction process. The feasibility of the clinical and echocardiographic variables measurement was assessed alongside preliminary statistical analyses including descriptive statistics and univariable LR, thereby informing variable selection and sample size calculation for a future, adequately powered multicentre study in which formal multivariable model construction and internal validation can be undertaken. By systematically evaluating RV systolic, during diastole and global function, alongside pulmonary haemodynamic variables and PDA assessment at standardised postnatal timepoints, this study provides foundational data to inform the design of larger, appropriately powered studies to construct a BPD prediction model in the first 10 days after birth (DAB).

## 2. Materials and Methods

### 2.1. Study Design and Setting

A single-centre prospective feasibility study was undertaken at a tertiary neonatal intensive care unit (NICU) as part of the REPORT-BPD project. The protocol received ethical approval from the UK Health Research Authority (IRAS ID: 311906). All procedures conformed to the Declaration of Helsinki, and parental or guardian written consent was secured for each participant [29].

### 2.2. Participants

The study recruited 40 preterm neonates delivered before 32 weeks of gestation. Full eligibility criteria, including inclusion and exclusion criteria, and recruitment procedures, have been previously described [29,30]. Briefly, eligible infants were those admitted to the neonatal unit with a clinical indication for echocardiography; those with significant congenital or chromosomal anomalies, poor prospect of survival, whose parents declined consent, or who were on blood pressure support medication, e.g., inotropes, were excluded [29,30]. Recruitment was completed in 11 months (June 2022–May 2023), seven months ahead of the 18-month period specified in the study protocol. Further details, for example, the number of preterm infants screened and the recruitment rate, are included in a previous publication of the REPORT-BPD study feasibility outcomes [30]. All infants underwent neonatologist-performed echocardiography (NPE) at two timepoints: Scan 1 (5th day after birth (DAB)) and Scan 2 (9th DAB) [29,30].

### 2.3. Echocardiographic Assessment

Cardiac imaging was carried out at the bedside by a trained neonatologist using either a Philips CX50 or EPIQ 7 platform (Philips Healthcare, Bothell, WA, USA) with a 12 MHz probe. A uniform scanning protocol, informed by established international recommendations, was followed throughout [31,32]. Subsequent offline analysis of acquired images and measurements was completed using TomTec cardiac analysis software (TomTec-ARENA, version TTA2.42; TomTec Imaging Systems GmbH, Unterschleißheim, Germany). Whenever possible, cardiac echocardiographic variables, e.g., tricuspid annular plane systolic excursion (TAPSE) or tissue Doppler imaging (TDI) velocities, were normalised to the interventricular septal (IVS) length measured at the end of the diastolic phase of the cardiac cycle. Cardiac cycle phases were confirmed by the opening and closure of the cardiac valves and through the ECG recordings in the echo scans [29]. Tricuspid regurgitation (TR) peak velocity (Vmax) and the derived peak pressure gradient (PGmax) were measured from continuous-wave Doppler interrogation of the TR jet, in accordance with the American Society of Echocardiography guidelines for targeted neonatal echocardiography [33]. A complete TR spectral envelope was required for reliable measurement; where only a partial envelope was obtainable, the measurement was recorded as such and flagged, given the expected underestimation inherent to incomplete spectral sampling.

To minimise the risk of E and A wave fusion, echocardiographic acquisitions were performed during periods of behavioural quiescence when heart rates were relatively lower [31,34]. Multiple cardiac cycles were acquired, and measurements were obtained from cycles with the clearest E-A wave separation [31,34]. The RV FAC was measured using offline speckle tracking analysis with TomTec 2D Cardiac Performance Analysis software (2D CPA, version 1.4.0.156; TomTec Imaging Systems, Germany), from the apical four-chamber view.

### 2.4. BPD Definition

Bronchopulmonary dysplasia was diagnosed using the same definition as previously reported in the feasibility outcomes analysis [30], integrating the criteria of Jobe and Bancalari (2001) and Higgins et al. (2018) [4,35]. The latter addresses limitations of the former in classifying infants receiving humidified heated high-flow nasal cannula therapy or those who die before 36 weeks postmenstrual age, thereby permitting a comprehensive assessment of all included preterm infants [4,35].

### 2.5. Statistical Analyses

All statistical analyses were performed using IBM SPSS Statistics (Version 29.0; IBM Corp., Armonk, NY, USA). The distributions of all continuous variables were visualised using histograms and box plots. Given the limited sample size and non-normal distribution of several variables, non-parametric methods were employed.

Descriptive statistics are presented according to BPD status. Between-group comparisons were performed using the Mann–Whitney U (MWU) test. Univariable binary LR was performed for each candidate variable, yielding odds ratios (OR) with 95% confidence intervals (CI).

Candidate variables were selected a priori based on previous studies for the clinical variables [18] and physiological rationale for the echocardiographic variables. Variables were organised into six domains reflecting the multifaceted pathogenesis of BPD:

1. Clinical (three variables): Gestational age, current weight, and mean airway pressure (MAP), as these are established baseline risk factors and markers of disease severity.
2. RV Systolic Function (five variables): RV s’ (absolute and normalized), TAPSE (absolute and normalized), and RV FAC reflecting longitudinal and global systolic contraction. For prediction model consideration, only the normalised forms of RV s’ and TAPSE, together with RV FAC, were to be carried forward (three variables).
3. RV Function during diastole (eleven variables): RV e’ (absolute and normalized), RV a’ (absolute and normalized), tricuspid valve (TV) E (absolute and normalized), TV A (absolute and normalized), RV E/e’ ratio, RV e’/a’ ratio and TV E/A ratio, characterizing ventricular relaxation and filling dynamics.
4. RV Global Function (two variables): Isovolumic relaxation time (IVRT) and isovolumic contraction time (IVCT)—reflecting overall myocardial efficiency.
5. Pulmonary Vascular Status (six variables): RV myocardial performance index (MPI) by pulsed wave (PW) Doppler and TDI, tricuspid regurgitation (TR) peak gradient (retained as a candidate for future evaluation), pulmonary artery acceleration time (PAAT), RV ejection time (RVET), and PAAT/RVET ratio variables reflecting pulmonary haemodynamics and vascular resistance.
6. The PDA Severity Assessment (one variable): No formal clinically validated PDA scoring system was applied in the present study, as the development of such a tool was beyond the scope of this work. Nevertheless, several PDA-related echocardiographic indices were examined, including absolute and normalised PDA diameter (PDA/IVS ratio), left atrium to aortic root (LA:Ao) ratio, left pulmonary artery diastolic velocity, PDA diastolic velocity, mitral valve (MV) E/A ratio, and anterior cerebral artery (ACA) diastolic flow pattern. These indices were compared between preterm infants with and without BPD at both echocardiographic timepoints.

Accordingly, the REPORT-BPD feasibility study assessed 26 candidate variables across six clinical and echocardiographic domains. The primary analyses were limited to descriptive statistics for all candidate variables and univariable LR to characterise the behaviour of individual variables in this cohort, thereby generating preliminary data to inform the design and sample size requirements for a definitive multicentre model development and internal validation study. Where *p*-values are reported, no adjustment for multiple comparisons was applied given the hypothesis-generating objectives; associations are interpreted as candidate variables for future validation rather than definitive independent predictors.

Receiver Operating Characteristic (ROC) curves were generated based on established physiological and clinical assumptions underpinning the behaviour of each variable in relation to BPD. For clinical variables, it is well recognised that lower gestational age and lower birth weight are associated with a higher risk of developing BPD, with the incidence inversely proportional to both parameters [36,37]. Accordingly, ROC curves for these variables were generated on the assumption that smaller values are associated with a greater likelihood of BPD. The same directional principle was applied to echocardiographic variables, guided by known pathophysiological relationships. PDA is well established as a risk factor for BPD, with evidence demonstrating that a longer exposure to a significant ductal shunt independently increases the odds of BPD [38]. Several echocardiographic indices reflecting left heart volume overload secondary to a significant left-to-right PDA shunt were therefore assessed on the assumption that larger values are associated with BPD. For example, the MV E/A ratio is recognised as a marker of raised left atrial loading in the context of a significant PDA; in preterm infants, the MV E/A ratio is typically <1, but reverses to greater than 1 in the presence of elevated left cardiac overloading resulting from a PDA [39,40]. Given that PDA is associated with BPD, a higher MV E/A ratio, as a surrogate marker of PDA, was therefore assumed to be associated with a greater likelihood of BPD, and the ROC curve was generated accordingly.

## 3. Results

**Figure 1:**
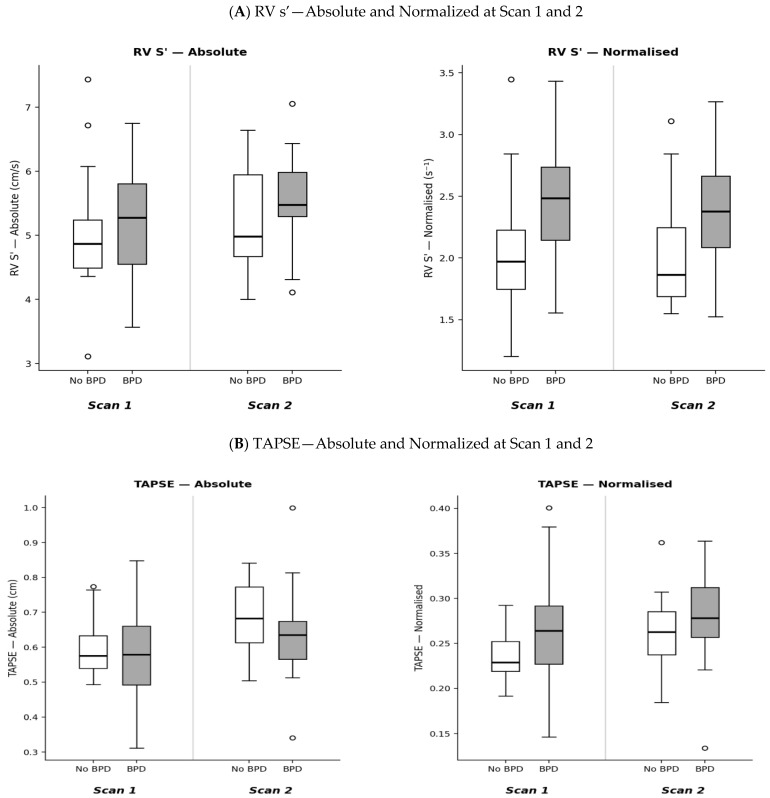

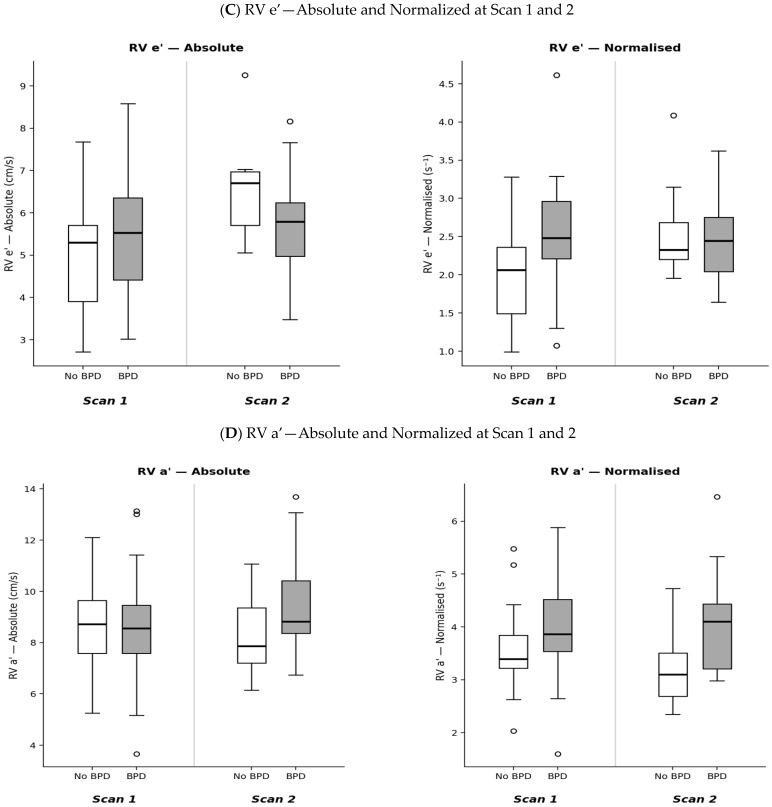

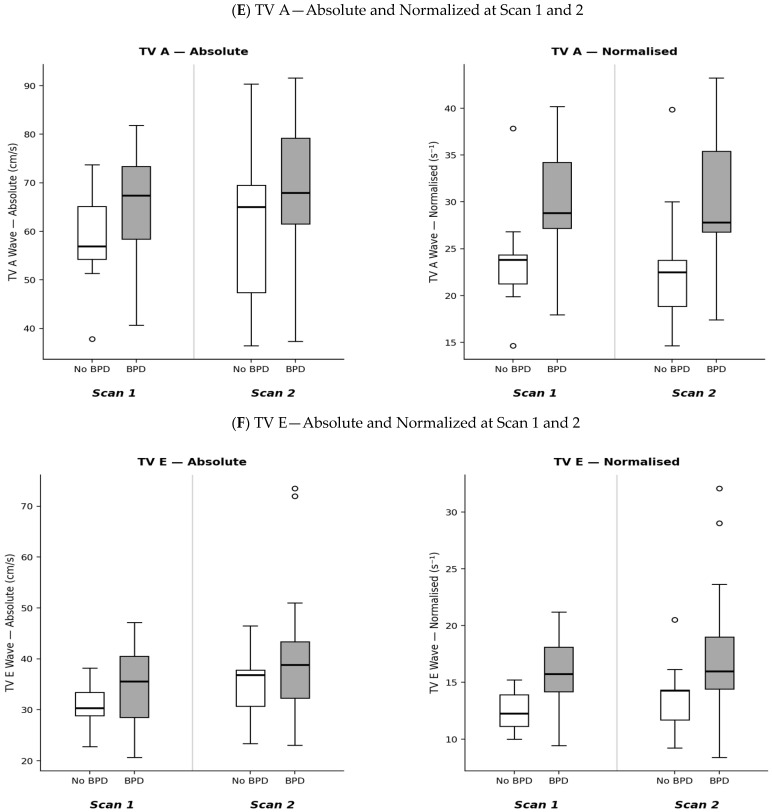
Between-group comparisons of absolute and normalized echocardiographic measurements at Scan 1 (Day 5) and Scan 2 (Day 9) in preterm infants with and without BPD. BPD = bronchopulmonary dysplasia; RV = right ventricle; s’ = systolic tissue velocity; e’ = early diastolic tissue velocity; a’ = late diastolic tissue velocity; TAPSE = tricuspid annular plane systolic excursion; TV = tricuspid valve; E = early diastolic inflow velocity; A = late diastolic inflow velocity.

Forty preterm infants were enrolled in the study, of whom 27 (68%) were subsequently diagnosed with BPD and 13 (32%) were not. One infant in the BPD group was lost to follow-up prior to the second echocardiographic assessment, resulting in 39 infants available for analysis at Scan 2. Both echocardiographic evaluations were well-tolerated, with no adverse events reported [30]. Box plots comparing absolute and normalised cardiac indices are shown in Figure 1A–F. Descriptive statistics for the candidate variables at both timepoints are presented in Table 1, Table 2, Table 3, Table 4 and Table 5, with corresponding ROC graphs in Appendix A and Appendix B.

### 3.1. Clinical Variables (Appendix A.1, Figure A1; Appendix A.2) (Table 1 and Table 2)

Clinical variables demonstrated high discriminatory ability for BPD. Preterm infants with BPD were born at lower gestational ages than those who did not (OR 0.50 per week, 95% CI 0.33–0.76, MWU *p* < 0.001). Current weight at assessment was similarly discriminative (OR 0.54 per 100 g, 95% CI 0.36–0.80, MWU *p* < 0.001).

The MAP demonstrated a good discriminatory ability, too. At Scan 1, preterm infants in the BPD group required higher MAP than those who did not (OR 1.86 per 1 cmH_2_O, 95% CI 1.25–2.78, MWU *p* < 0.001). By Scan 2, most infants without BPD had been successfully weaned off respiratory support (median 0.0, IQR 0.0–0.0 cmH_2_O), whilst those who developed BPD remained on respiratory support (OR 1.74 per 1 cmH_2_O, 95% CI 1.26–2.40, MWU *p* < 0.001).

### 3.2. RV Systolic Function Variables (Appendix A.1, Figure A2; Appendix A.2) (Table 1 and Table 2)

Assessment of RV systolic function revealed the importance of normalisation for identifying differences between groups (Table 1 and Table 2). Absolute RV systolic velocity (RV s’) did not differ statistically between groups at either timepoint (Scan 1: OR 1.24 per 1 cm/s, 95% CI 0.59–2.61, MWU *p* = 0.45; Scan 2: OR 1.65 per 1 cm/s, 95% CI 0.65–4.22, MWU *p* = 0.26). However, when normalised for IVS length, RV s’ was elevated in the BPD group at both assessments. At Scan 1, normalized RV s’ was elevated in the BPD group (OR 2.26 per 0.5 s^−1^, 95% CI 1.03–4.96, MWU *p* = 0.02). This difference persisted at Scan 2 (OR 2.76, 95% CI 1.11–6.89, MWU *p* = 0.01) (Figure 1A).

The TAPSE, both absolute and normalised, showed no significant differences between groups at either timepoint (all MWU *p* > 0.15). On univariable LR, absolute TAPSE yielded ORs below unity at both timepoints (Scan 1: OR 0.89 per 0.1 cm increase, 95% CI 0.51–1.56, *p* = 0.69; Scan 2: OR 0.78 per 0.1 cm increase, 95% CI 0.45–1.38, *p* = 0.39), suggesting a trend toward lower excursion in the BPD group. However, following normalisation to IVS length, this direction reversed, with odds ratios exceeding unity (Scan 1: OR 3.29, 95% CI 0.71–15.30, *p* = 0.13; Scan 2: OR 1.95, 95% CI 0.46–8.27, *p* = 0.36), though neither reached statistical significance (Figure 1B). The RV FAC measured using speckle tracking analysis showed no significant between-group differences at either timepoint (Scan 1: OR 0.95, 95% CI 0.86–1.06; Scan 2: OR 0.98 per 1% change, 95% CI 0.90–1.06).

### 3.3. RV Function During Diastole Variables (Appendix A.1, Figure A3; Appendix A.2) (Table 1 and Table 2)

At Scan 1, absolute RV e’ was marginally elevated in the BPD group, whilst normalised RV e’ was higher in the BPD group and reached statistical significance on MWU testing (OR 1.75 per 0.5 s^−1^, 95% CI 0.98–3.13, MWU *p* = 0.03). At Scan 2, absolute RV e’ was lower in the BPD group (OR 0.50 per 1 cm/s, 95% CI 0.24–1.02). In contrast, normalised values showed no statistical difference (OR 0.91 per 0.5 s^−1^, 95% CI 0.49–1.71) (Figure 1C).

At Scan 1, absolute RV a’ showed no statistical difference (OR 0.93 per 1 cm/s, 95% CI 0.67–1.30), yet normalised values were higher in the BPD group (OR 1.24 per 0.5 s^−1^, 95% CI 0.85–1.80). At Scan 2, absolute RV a’ had an OR of 1.43 (95% CI 0.92–2.22), while the normalised value reached statistical significance (OR 1.93, 95% CI 1.13–3.28) (Figure 1D).

Normalised TV inflow velocities demonstrated consistent differences between groups at both timepoints. Normalised TV A velocity was elevated in the BPD group at Scan 1 (OR 1.32 per 1 s^−1^, 95% CI 1.08–1.62). This difference persisted at Scan 2 (OR 1.23 per 1 s^−1^, 95% CI 1.05–1.45). Similarly, normalised TV E velocity was higher in the BPD group at Scan 1 (OR 1.60, 95% CI 1.15–2.23) and remained elevated at Scan 2 (OR 1.26 per 1 s^−1^, 95% CI 1.00–1.59) (Figure 1E,F).

The RV E/e’ ratio, reflecting filling pressure, was elevated in the BPD group at Scan 2 (OR 2.03, 95% CI 1.06–3.88), but not at Scan 1 (OR 1.01, 95% CI 0.80–1.29). The TV E/A ratio did not differ between groups at Scan 1 (OR 0.94 per 0.1, 95% CI 0.49–1.81). By Scan 2, the same pattern was demonstrated with no statistical difference (OR 0.90 per 0.1, 95% CI 0.55–1.46).

The RV e’/a’ ratio demonstrated no significant difference between groups at Scan 1 (OR 1.16, 95% CI 0.86–1.56) but was lower in the BPD group at Scan 2 (OR 0.54, 95% CI 0.32–0.90), with both ORs expressed per 0.1 increase.

### 3.4. RV Global Function (Appendix A.1, Figure A4; Appendix A.2) (Table 1 and Table 2)

The RV isovolumic relaxation time (IVRT) showed no significant differences between groups at either timepoint (Scan 1: OR 1.01, 95% CI 0.96–1.06; Scan 2: OR 0.99 per millisecond (ms), 95% CI 0.95–1.04). Similarly, RV isovolumic contraction time (RV IVCT) did not differ between groups (Scan 1: OR 0.98, 95% CI 0.94–1.02; Scan 2: OR 0.97 per ms, 95% CI 0.92–1.02).

### 3.5. Pulmonary Vascular Status Variables (Appendix A.1, Figure A5; Appendix A.2) (Table 1 and Table 2)

Variables reflecting pulmonary haemodynamics demonstrated important associations with BPD, particularly at the earlier timepoint. The RV MPI-PW was elevated in the BPD group at Scan 1 (OR 2.28 per 0.1 increase, 95% CI 1.15–4.54). This difference was attenuated by Scan 2 (OR 1.37, 95% CI 0.91–2.08). In contrast, RV MPI-TDI showed no association at either timepoint (Scan 1: OR 1.00, 95% CI 0.70–1.42; Scan 2: OR 0.80 per 0.1 increase, 95% CI 0.45–1.41).

Among pulmonary vascular indices, a complete TR spectral envelope was obtainable in 35% (14/40) of infants at Scan 1 and 18% (7/39) at Scan 2, giving a combined obtainability rate of 27% across both timepoints. Where only a partial envelope was obtainable, measured values were consistently lower than those derived from complete envelopes.

The PAAT was reduced in the BPD group at Scan 1 (OR 0.72 per 10 ms, 95% CI 0.51–1.00), but this difference was no longer statistically significant at Scan 2 (OR 0.87 per 10 ms increase, 95% CI 0.64–1.19).

The RVET was shorter in the BPD group at both timepoints (Scan 1: OR 0.49, 95% CI 0.27–0.89; Scan 2: OR 0.61 per 10 ms, 95% CI 0.38–0.97), while the PAAT/RVET ratio did not reach statistical significance at either timepoint (Scan 1: OR 0.61, 95% CI 0.33–1.15; Scan 2: OR 0.94 per 0.1 increase, 95% CI 0.54–1.64).

### 3.6. PDA-Related Variables (Appendix B, Figure A6, Figure A7, Figure A8, Figure A9 and Figure A10) (Table 3, Table 4 and Table 5)

At Scan 1, BPD-affected preterm infants had a larger normalised PDA diameter (median 0.09 [IQR 0.08–0.12] vs. 0.06 [IQR 0.01–0.07]) and a larger absolute PDA diameter (median 0.21 cm [IQR 0.18–0.24] vs. 0.13 cm [IQR 0.04–0.18]). On univariable LR, each 0.01 increase in normalised PDA diameter was associated with increased odds of BPD (OR 1.70, 95% CI 1.21–2.39), while each 0.01 cm increase in absolute PDA diameter was associated with an OR of 1.21 (95% CI 1.07–1.38).

At Scan 1, the LPA diastolic velocity was higher in the BPD group (OR 1.63 per 0.1 m/s, 95% CI 1.06–2.51). The remaining PDA-related variables did not differ between the groups at this timepoint: LA:Ao ratio (OR 1.07 per 0.1 increase, 95% CI 0.86–1.33), PDA diastolic velocity (OR 1.05 per 0.1 m/s increase, 95% CI 0.94–1.16), and MV E/A ratio (OR 1.04 per 0.1 increase, 95% CI 0.75–1.45).

At Scan 2, normalised PDA diameter remained higher in the BPD group (OR 1.29 per 0.01 increase, 95% CI 1.04–1.60), and LA:Ao ratio emerged as a differentiator between groups (OR 1.31 per 0.1 increase, 95% CI 1.02–1.68). Absolute PDA diameter showed a trend toward larger values in the BPD group (MWU *p* = 0.049) but did not reach significance on LR (OR 1.09 per 0.01 cm increase, 95% CI 1.00–1.18). LPA diastolic velocity (OR 1.22 per 0.1 m/s increase, 95% CI 0.88–1.69), PDA diastolic velocity (OR 1.02 per 0.1 m/s increase, 95% CI 0.92–1.13), and MV E/A ratio (OR 0.86 per 0.1 increase, 95% CI 0.61–1.22) did not differ between groups at this timepoint.

All preterm infants with reversed ACA diastolic flow in the context of a confirmed left-to-right ductal shunt developed BPD at both timepoints (Scan 1: n = 3; Scan 2: n = 4). Among preterm infants with absent ACA diastolic flow, 2 of 3 developed BPD at Scan 1; no preterm infant had absent ACA diastolic flow at Scan 2. Among those with antegrade ACA diastolic flow, 22 of 34 developed BPD at Scan 1 and 22 of 35 at Scan 2.

Candidate variables for inclusion in future prediction model construction are summarised in Table 6.

## 4. Discussion

The observed BPD incidence of 68% is consistent with published rates for extremely preterm infants [2,41], reflecting the gestational distribution of this cohort, which was enriched for extremely preterm infants.

As the haemodynamic processes at the early stages of the evolving BPD are a result of complex interactions between the right side of the heart and pulmonary vascular bed [42,43], the selection of candidate variables incorporated clinical variables with established associations with BPD, complemented by echocardiographic indices or variables affording holistic assessment of RV function across all phases of the cardiac cycle; systolic, diastolic, and global, as well as variables interrogating the haemodynamic relationships between right ventricular performance, pulmonary vascular status, and PDA shunt.

### 4.1. Proposed Variables’ Groups for BPD Prediction Model (Table 6)

Group 1: Clinical Variables

Clinical factors represent the baseline risk profile and physiological stress at the time of assessment. These variables are readily available and provide context for interpreting echocardiographic findings. Lower gestational age and birth weight are established risk factors for BPD [2,30,36]. The MAP is a reflection of the degree of respiratory support required, serving as an early marker of disease severity and lung injury at the time of echocardiographic assessment [30].

Clinical variables demonstrated the strongest discriminatory ability for BPD among all domains evaluated. Our findings are consistent with previous literature showing that lower gestational age and lower weight, whether birth weight [30] or current weight, as reported in this study, are associated with BPD, reflecting the inherent vulnerability of the immature lung to injury. The MAP also exhibited a good discriminatory performance at both timepoints, with preterm infants who developed BPD requiring higher MAP at Scan 1, whilst most preterm infants without BPD were successfully weaned of respiratory support by Scan 2. These findings are consistent with the existing literature and support the inclusion of clinical variables alongside echocardiographic indices in future BPD prediction models [16,44].

Group 2: Right Ventricular (RV) Systolic Function

RV systolic function reflects the heart’s ability to maintain cardiac output against the pulmonary vascular bed. Impaired RV systolic function may indicate increased pulmonary vascular resistance associated with evolving lung disease [45].

The RV s’ was elevated in the BPD group at both timepoints. Absolute RV s’ was higher in the BPD group at Scan 1 and Scan 2, though neither reached statistical significance. Notably, the OR increased between timepoints, suggesting a trend toward greater separation in systolic velocity as the early postnatal period progressed. Following normalisation, this trend became more apparent, with the OR rising further and reaching statistical significance at both timepoints. The amplification of OR from absolute to normalised values, and from Scan 1 to Scan 2, suggests that the augmented systolic phase in the BPD group might be an indication of the RV response to the increase in PVR, i.e., afterload rise.

Absolute TAPSE was similar between groups at Scan 1 and marginally lower in the BPD group at Scan 2, though neither reached statistical significance. Following normalisation to IVS length, the direction of OR was reversed, with higher values observed in the BPD group at both timepoints. Although neither the absolute nor normalised TAPSE values reached statistical significance, the consistent reversal of the OR direction across both timepoints represents an observation of potential physiological interest. TAPSE is recognised as an afterload-dependent measure of RV longitudinal function, and its values are influenced by changes in PVR [46]. Our findings showed that the absolute TAPSE values comparing groups with and without BPD are similar to the results of Neumann et al. (2021), which demonstrated that TAPSE was decreased in preterm infants who developed BPD compared to those without BPD [47]. However, normalisation of TAPSE to IVS length produced higher values in preterm infants with BPD at both timepoints, suggesting that the trend toward lower absolute excursion in the BPD group could be a reflection of smaller cardiac dimensions rather than impaired longitudinal RV systolic function, a finding consistent with the hypothesis of a compensatory hyperdynamic RV systolic response during the early evolution of BPD in preterm infants. The RV FAC showed no significant difference between groups at either timepoint.

In the early postnatal period, preterm infants who subsequently develop BPD are likely exposed to rising PVR before overt RV failure has had time to manifest. During this compensated phase, the RV may mount a hyperdynamic response to maintain cardiac output against increased afterload, and normalisation to IVS length by accounting for the smaller cardiac dimensions of more premature infants may unmask this compensatory effort, which absolute values alone obscure.

The behaviour of both normalised RV s’ and normalised TAPSE is consistent with this interpretation, as both indices demonstrated higher values in the BPD group following normalisation. This compensatory hyperdynamic RV response to the elevated PVR associated with early BPD may also account for the absence of discriminatory findings when comparing RV FAC between groups, as global measures of RV function may remain preserved during this early adaptive phase, thereby limiting their ability to differentiate between infants with and without evolving BPD. Hence, three variables, normalised RV s’, normalised TAPSE, and RV FAC, will be considered as candidate indices for inclusion in the construction of an early BPD prediction model.

Group 3: RV Function in Diastole

RV diastolic function reflects myocardial relaxation and filling properties.

The indices of RV function in diastole demonstrated complex patterns that highlight the potential value of normalisation and the evolving nature of cardiac adaptation over time.

Early diastolic tissue velocity (RV e’) showed variable patterns across timepoints. At Scan 1, normalised RV e’ was higher in the BPD group, whilst absolute values showed no statistical difference. This elevation likely reflects enhanced early diastolic recoil secondary to the compensatory systolic response to pulmonary vascular haemodynamic changes in early BPD. At Scan 2, whilst normalised RV e’ remained higher in the BPD group on the box plot (Figure 1C), the difference was no longer statistically significant. This narrowing of the gap may reflect two concurrent processes: normal diastolic maturation in the No BPD group, with normalised e’ rising from 2.1 to 2.3, and attenuation of the initial compensatory recoil in the BPD group, with normalised e’ stagnating from 2.5 to 2.4, likely reflecting compromised myocardial maturation as BPD evolves or which could be due to different changes in preload between groups. In contrast, absolute RV e’ at Scan 2 was lower in the BPD group, emphasising the potential value of accounting for cardiac size when interpreting diastolic function.

The TV E wave, which reflects early passive filling across the tricuspid valve [48], showed a complementary but distinct pattern. Absolute TV E velocity did not differ between groups at either timepoint; however, the normalised value was higher in the BPD group at Scan 1 and Scan 2. The persistent elevation of normalised TV E velocity in the BPD group, even as normalised RV e’ stagnated, is noteworthy: it suggests that higher filling velocities are being driven into a ventricle whose myocardial relaxation is no longer keeping pace and points towards worse RV filling properties.

The higher normalised RV a’ observed in the BPD group at both timepoints, with a widening difference by Scan 2, suggests greater myocardial motion during atrial systole. RV a’ is a TDI measure of myocardial velocity at the tricuspid annulus during atrial contraction and serves as an indirect marker of atrial contribution to ventricular filling. A more direct measure is the TV A velocity obtained by PW Doppler, which reflects actual blood flow across the TV during atrial contraction [48]. Interestingly, normalised TV A velocity was higher in the BPD group at both timepoints, providing confirmatory evidence of increased atrial contribution to RV filling, while absolute TV A velocity differed only at Scan 1 and not at Scan 2.

The E/e’ ratio estimates RV filling pressures, with higher values indicating elevated filling pressures. In normally maturing preterm infants, myocardial velocities increase with postnatal age while the E/e’ ratio decreases, reflecting the maturation of diastolic function [49]. This ratio was calculated from absolute values, as normalising both numerator and denominator by the same metric would cancel out the effect of normalisation. However, normalised values of the individual components remain informative as explained above. In the present study, preterm infants without BPD followed this expected trajectory of the normal maturation pattern (E/e’ ratio 6.2 → 5.3), whereas the BPD group demonstrated a rising E/e’ ratio (6.5 → 6.8). While the two groups did not differ statistically at Scan 1, a difference had emerged by Scan 2, which may reflect the divergent trajectories of diastolic maturation between the groups. Physiologically, RV diastolic filling depends on recoil, relaxation, and stiffness; the higher normalised RV s’ in the BPD group would be expected to enhance recoil and lower E/e’. The opposite finding suggests that impaired relaxation and increased stiffness outweighed the recoil benefit, indicating that diastolic dysfunction may be more pronounced than the E/e’ ratio in isolation suggests.

The RV e’/a’ ratio, which was not normalised since it is a ratio, further supports the pattern of divergent diastolic maturation. At Scan 1, the e’/a’ ratio did not differ between groups. By Scan 2, however, a difference had emerged. The No BPD group demonstrated a normal maturational increase in e’/a’ ratio (0.60 → 0.83), reflecting the expected developmental shift from atrial contraction-dependent filling towards early diastolic relaxation-predominant filling. In contrast, the BPD group remained essentially static (0.63 → 0.64), indicating failure of normal diastolic maturation.

The TV E/A ratio, which also uses absolute measurements, did not differ between groups at either timepoint. Both groups demonstrated A-wave dominant TV inflow (E/A < 1), consistent with the physiological pattern of neonatal diastolic filling in which ventricular compliance is inherently limited and atrial contraction contributes a proportionally greater share of RV filling than in the mature heart [50]. Nevertheless, the trajectories of the two groups differed subtly: the No BPD group showed a modest maturational increase in TV E/A ratio (0.56 → 0.61), suggesting early progression towards relaxation-predominant filling, whereas the BPD group remained essentially static (0.55 → 0.56). Although this difference did not reach statistical significance, the directional trend is concordant with the other diastolic indices, stagnating normalised RV e’ and rising E/e’ ratio, collectively suggesting impaired diastolic maturation in infants with evolving BPD [51,52].

Whilst the normalisation of diastolic phase variables such as TV E and A wave velocities and TDI-derived RV e’ and a’ to cardiac size is not routinely applied [51], the findings of this study suggest potential value in this approach. Comparative evaluation of both normalised and absolute measurements in a larger cohort of preterm infants is warranted.

Group 4: RV Global Function

Global function variables integrate systolic and diastolic performance, providing an overall assessment of RV efficiency. Prolonged, for example, IVRT, suggests impaired relaxation, which may occur with increased afterload or intrinsic myocardial dysfunction. IVRT is inversely correlated with cardiac index and has been validated in pulmonary artery systolic pressure assessment [53].

The RV IVRT remained essentially static in both groups between scans. The apparent stability of IVRT in the BPD group may reflect a pseudo-normal phenomenon driven by two opposing haemodynamic forces. On the one hand, impaired myocardial relaxation as evidenced by the stagnating normalised RV e’ in the BPD group (2.5 → 2.4), compared with the expected maturational rise observed in the No BPD group (2.1 → 2.3), would be expected to prolong IVRT, as the ventricle takes longer to reduce its pressure below right atrial pressure and allow TV opening. On the other hand, concurrently rising right atrial pressure in the BPD group, as suggested by the increasing E/e’ ratio (6.5 → 6.8) and persistently elevated TV A velocities, would promote earlier TV opening by increasing the pressure gradient on the atrial side, effectively shortening IVRT [48,51]. In other words, the hypothesis is that these two opposing pathophysiological processes may neutralise one another, yielding a pseudo-normal and apparently stable IVRT [48,51].

The RV IVCT also showed no statistical significance between groups, but the trajectories between scans differed. The group without BPD demonstrated shortening (47 → 31 ms), which may be consistent with normal postnatal reduction in PVR, lowering the pressure threshold for PV opening [54]. The BPD group had a shorter IVCT at Scan 1 (36 ms) despite presumably higher PVR, which we hypothesise reflects an enhanced rate of RV pressure generation consistent with the hyperdynamic systolic response observed in other indices, such as RV s’ and TAPSE.

Group 5: Pulmonary Vascular Status

Pulmonary vascular variables directly assess the pulmonary circulation, which is central to BPD pathophysiology. Elevated PVR and pressure are both consequences of and contributors to ongoing lung injury [24]. The RV MPI represents a distinctive echocardiographic index that integrates both systolic and diastolic components of RV function, thereby providing a comprehensive haemodynamic correlate with the evolving pulmonary vascular load. Its non-invasive nature and sensitivity to increasing pulmonary arterial pressure position it as a potentially valuable tool for early detection and longitudinal assessment of pulmonary haemodynamic derangements in neonatal populations [55].

Several variables were evaluated as potential markers of pulmonary haemodynamics.

Findings at the earliest timepoint indicated the likely presence of evolving pulmonary vascular disease: PAAT was shorter in the BPD group at Scan 1, and RVET was reduced at both timepoints. By Scan 2, PAAT had increased in both groups, consistent with the expected postnatal fall in PVR, but the between-group difference was no longer significant, possibly due to the wide variance in the BPD group (IQR 47–85).

As reported by previous research, the clinical utility of the peak TR pressure gradient can often be constrained by the frequent inability to detect a regurgitant jet in preterm infants [24,56]; in the present study, a complete TR spectral envelope was obtainable in only a minority of scans, and the majority of partial envelopes yielded low-velocity TR signals. The TR peak gradient was therefore not included in statistical analysis due to the low obtainability rate of complete spectral envelopes.

Building on this observation, the obtainability rate of a complete TR jet envelope in the present cohort is consistent with previously reported rates in preterm populations, notably those reported by Seo and Choi in a mixed cohort of preterm infants with and without BPD [57]. This reflects a well-recognised limitation of TR-based pulmonary pressure estimation in extremely preterm infants, where small cardiac dimensions and frequent absence of an adequate regurgitant jet limit Doppler interrogation [24]. These findings indicate that the feasibility of obtaining complete TR spectral envelopes in extremely preterm infants requires prospective evaluation in a larger multicentre cohort before TR-based measurements can be considered suitable for inclusion in a clinically applicable BPD prediction model. In view of these feasibility constraints, echocardiographic indices that do not depend on the TR jet become particularly relevant.

PAAT was shorter in the BPD group at both timepoints. This is consistent with previous research indicating that shortened PAAT is a validated variable of elevated PVR, arterial pressure, and reduced pulmonary vascular compliance in preterm infants [56,58,59], and its presence as early as postnatal day seven has been shown to be associated with the subsequent development of BPD and late PH [24]. The finding of shortened PAAT in the BPD group in the present study suggests that pulmonary vascular changes in evolving BPD can be detectable in the early postnatal period, before the clinical manifestation of BPD [60].

The PAAT/RVET ratio showed a similar pattern: the BPD group trended lower at Scan 1, but this did not reach statistical significance, and the difference had resolved by Scan 2. The attenuation of the PAAT/RVET difference at Scan 2 contrasts with the persistent RVET reduction, suggesting that RVET shortening in the BPD group may have partly contributed to no difference in the ratio even when afterload remains elevated [61,62].

The concurrent elevation of RV MPI-PW at Scan 1 provides further support for the presence of increased RV afterload during this early period. RV MPI reflects the relationship between isovolumic time intervals and ejection time, with higher values indicating increased global ventricular workload in the context of elevated PVR [63,64]. The BPD group median remained unchanged at Scan 2, suggesting a persistent afterload burden even as the between-group difference fell below conventional significance, likely reflecting the larger variance in the BPD group at this timepoint (IQR 0.17–0.47).

Notably, RV MPI-TDI did not differ between groups at either timepoint. This discrepancy likely relates to the methodological differences between the two approaches: RV-MPI-PW requires measurement across two separate Doppler cycles (RV inflow and outflow), making it sensitive to beat-to-beat variation in heart rate, while RV-MPI-TDI derives all time intervals from a single myocardial velocity trace at the tricuspid annulus [34,65]. Although both indices reflect the same underlying cardiac time intervals and would in principle be expected to change in the same direction in response to alterations in PVR or contractility, they may differ in their ability to detect early haemodynamic changes in extremely preterm infants, and the divergent findings observed here warrant prospective comparative evaluation in a larger cohort.

RV Function–Pulmonary Vascular Change Coupling

Against this background of emerging pulmonary vascular remodelling, the normalised RV functional indices reveal a compensatory hyperdynamic RV systolic response. This is consistent with the compensatory phase of the RV pressure–afterload relationship, in which the RV enhances contractility to maintain cardiac output in the face of elevated PVR, before the transition to decompensation and failure [66]. The higher normalised RV e’ at both timepoints suggests that this rise in systolic contraction generates greater elastic recoil during early diastole, facilitating ventricular filling through the systolic–diastolic coupling mechanism [67]. The consistency of this pattern, shortened PAAT and RVET indicating high PVR, elevated RV MPI-PW reflecting increased global workload, with preserved RV-MPI-TDI suggesting that myocardial compensation remains intact, points toward a unified early adaptive RV response to the evolving pulmonary vascular changes that precede the clinical diagnosis of BPD. Whether these early haemodynamic findings can be used prospectively to identify preterm infants at risk of developing BPD, and whether they represent a window for targeted intervention before the transition from adaptive compensation to established BPD and RV dysfunction with or without PH, warrants investigation in larger studies.

Group 6: Patent Ductus Arteriosus Assessment

The PDA plays a significant role in neonatal cardiopulmonary haemodynamics. A PDA can contribute to pulmonary over-circulation and lung injury.

A persistent PDA with significant left-to-right shunting is a well-recognised contributor to the haemodynamic burden in preterm infants and may contribute to the pathogenesis of BPD [28,68].

Several patterns emerged among the PDA-related variables examined in our cohort. At Scan 1, absolute PDA diameter was larger in the BPD group, as was normalised PDA diameter, and LPA diastolic velocity was also higher in the BPD group. At Scan 2, normalised PDA diameter remained an important marker, and at this timepoint, the LA:Ao ratio emerged as a marker, whereas LPA diastolic velocity no longer differed between the groups. The PDA diastolic velocity and MV E/A ratio did not differ between groups at either timepoint. The shift in significance of individual PDA-related variables between timepoints may be an indication of the evolving haemodynamic consequences of ductal shunting. For example, the emergence of the LA:Ao ratio as a potential marker at Scan 2 is consistent with progressive left atrial dilatation from cumulative volume loading, as the sustained pulmonary over-circulation from a persistent left-to-right shunt begins to manifest as measurable chamber remodelling by the end of the first postnatal week.

However, incorporating multiple individual PDA-related echocardiographic variables into a BPD prediction model risks introducing multicollinearity and model instability [69]. PDA severity scoring systems have been developed to address this aspect by condensing multiple echocardiographic variables of pulmonary over-circulation and systemic hypoperfusion into a single numerical value [70,71,72,73].

The choice of scoring system, however, warrants careful consideration. In our study, all preterm infants with reversed ACA diastolic flow at both timepoints in the context of a confirmed left-to-right ductal shunt developed BPD (Scan 1: n = 3; Scan 2: n = 4), suggesting that this marker of cerebral diastolic steal identifies a subgroup with the most haemodynamically consequential ductal shunting. Nonetheless, contemporary PDA scoring systems do not include ACA diastolic flow as a separate variable [70,71,72,73].

Although a formal cerebral perfusion-focused PDA (cPDA) scoring proposal is beyond the remit of this feasibility study and limited by the small sample, the occurrence of BPD among preterm infants with reversed ACA flow raises the question of whether cerebral perfusion markers warrant inclusion in future PDA severity assessment, driven by clinical relevance rather than statistical dominance. Reversed diastolic flow in the ACA represents direct evidence of cerebral steal: a clinically concerning phenomenon whereby blood is diverted away from the brain through a low-resistance ductal pathway (ductal steal) during diastole [74,75,76]. As the quantitative indices, such as the resistive index, have shown inconsistent associations with outcomes in statistical analyses [77,78], the qualitative assessment of ACA flow direction (positive, absent, or reversed) provides clinically actionable information that is immediately interpretable at the bedside.

Similarly, the proposal of applying the normalisation of the PDA diameter to the IVS length at the end of diastole was informed by clinical reasoning regarding the differential haemodynamic impact of a given ductal size across infants of varying cardiac dimensions [79]. Whilst absolute PDA diameter demonstrated strong statistical associations with BPD in this cohort, the normalised measure provides a more physiologically meaningful assessment that accounts for individual variation, a principle increasingly recognised in paediatric echocardiography [79,80]. Also, cardiac dimensions in preterm infants correlate closely with gestational age and gestational-age-appropriate body size [81,82], making a cardiac structure a physiologically appropriate comparator.

In contrast, normalisation to body weight, which is utilised by some of the current PDA scoring systems [72,73], introduces its own limitations in this population, particularly during the early postnatal period when extremely preterm infants undergo physiological contraction of the extracellular fluid compartment, resulting in significant weight fluctuations over the first postnatal week [83,84]. Furthermore, if birth weight is used as the index for normalisation, this may not accurately be representative of the cardiac dimensions in infants with abnormal intrauterine growth; growth-restricted preterm infants demonstrate relative cardiac hypertrophy and elevated left ventricular output when indexed to body weight [85,86], while large-for-gestational-age infants may have adiposity-related discordance between body weight and cardiac size [87]. Therefore, future research is needed to evaluate whether cardiac size-based normalisation is comparable or potentially superior in validity to currently employed approaches such as body weight-based normalisation.

Collective Summary: The identification of candidate echocardiographic and clinical variables represents a foundational step toward the final selection of variables for inclusion in a prediction model for BPD. Future work will focus on the prospective development of an early BPD prediction model integrating key markers reflecting the multifaceted pathophysiology of BPD; this approach aims to enable risk stratification and the early identification of preterm infants at highest risk when preventive or targeted therapeutic strategies may be most effective. Furthermore, the application of normalised rather than absolute echocardiographic measurements, where applicable, proved helpful in revealing pathophysiological differences between groups of preterm infants with and without BPD.

Moreover, such early risk stratification would enable the selective recruitment of high-risk preterm infants into the urgently needed interventional studies evaluating novel therapies [88,89]. In so doing, this would improve future research efficiency, reduce the exposure of low-risk infants to experimental interventions, and accelerate the translation of promising therapies into clinical practice.

### 4.2. Strengths

This study has several strengths. First, the early echocardiographic phenotyping across RV systolic, diastolic, and global function domains alongside pulmonary vascular status variables at two timepoints provides more details about the early postnatal period haemodynamic profile than studies focusing on a single domain or single timepoint. Second, the serial design enables trajectory analysis rather than cross-sectional comparison alone, revealing clinically meaningful patterns such as divergent maturation. Third, the normalisation of echocardiographic indices to cardiac size, and reporting both absolute and normalised values, adds transparency. Finally, the dual RV-MPI methodology (PW and TDI) in the same cohort uncovered a physiologically informative dissociation between elevated afterload and preserved myocardial compensation that would have been missed with either method alone.

### 4.3. Limitations

The limitations of this study are as follows: First, this was a single-centre feasibility study with a small sample size (n = 40). Second, data on treatments such as for PDA and the therapeutic use of postnatal corticosteroids were not collected, limiting the ability to adjust for these potential confounders. Third, the coexistence of a PDA with variable haemodynamic significance makes it difficult to attribute RV and pulmonary vascular changes solely to intrinsic pulmonary vascular disease. Fourth, PAAT and TR peak gradient are surrogate markers of pulmonary haemodynamics without invasive catheterisation validation, although the TR peak gradient obtainability rate is yet to be assessed in a larger trial. Finally, all NPE scans were conducted by a single operator, which ensures internal consistency but limits the assessment of inter-observer reproducibility and generalisability to other centres.

## 5. Conclusions

This study supports clinical and echocardiographic variables as candidates for inclusion in the construction of an early BPD prediction model. These domains provide the foundation for a comprehensive yet practical approach to BPD risk stratification. Model development and validation in a larger cohort are required to determine which variables, individually or in combination, offer optimal predictive accuracy for clinical application.

## Figures and Tables

**Table 1:** Descriptive statistics for candidate variables at Scan 1 (Day 5). Comparison between No BPD and BPD groups.

Parameter	N	No BPD (n = 13)					BPD (n = 27)					MWU *p*	OR	95% CI	LR *p*
		Mean	SD	SE	Median	IQR	Mean	SD	SE	Median	IQR				
***Clinical Variables***															
Gestational Age (weeks)	40	29	2.31	0.64	30	28–31	26	1.88	0.36	26	25–27	<0.001	0.50	0.33–0.76	<0.001
Current Weight (g)	40	1280	339	94	1200	1155–1544	796	228	44	763	632–967	<0.001	0.54	0.36–0.80	0.002
MAP (cmH_2_O)	40	3	3.3	0.9	3	0.0–4.0	8	2.7	0.5	8	5.9–10.4	<0.001	1.86	1.25–2.78	0.002
***RV Systolic Function***															
RV s’ (cm/s)—absolute	40	5.1	1.1	0.30	4.9	4.5–5.2	5.3	0.8	0.16	5.3	4.6–5.8	0.45	1.24	0.59–2.61	0.58
RV s’ (s^−^^1^)—normalised	40	2.1	0.58	0.16	2.0	1.8–2.2	2.5	0.46	0.09	2.5	2.2–2.7	0.02	2.26	1.03–4.96	0.04
TAPSE (cm)—absolute	40	0.60	0.09	0.02	0.6	0.5–0.6	0.58	0.13	0.03	0.6	0.5–0.7	0.56	0.89	0.51–1.56	0.69
TAPSE—normalised	40	0.24	0.03	0.01	0.23	0.22–0.25	0.27	0.06	0.01	0.26	0.23–0.29	0.16	3.29	0.71–15.30	0.13
RV FAC (%)	37	23	6.4	1.8	24	20–26	21	7.4	1.5	22	18–26	0.43	0.95	0.86–1.06	0.35
***RV Diastolic Function***															
RV e’ (cm/s)—absolute	40	5.0	1.43	0.40	5.3	3.9–5.7	5.4	1.35	0.26	5.5	4.4–6.4	0.42	1.23	0.74–2.02	0.42
RV e’ (s^−^^1^)—normalised	40	2.1	0.67	0.19	2.1	1.5–2.4	2.5	0.68	0.13	2.5	2.2–3.0	0.03	1.75	0.98–3.13	0.05
RV a’ (cm/s)—absolute	40	8.9	1.92	0.53	8.7	7.6–9.7	8.6	2.12	0.41	8.6	7.6–9.5	0.73	0.93	0.67–1.30	0.68
RV a’ (s^−^^1^)—normalised	40	3.6	0.97	0.27	3.4	3.2–3.8	4.0	0.95	0.18	3.9	3.5–4.5	0.17	1.24	0.85–1.80	0.26
RV E/e’ ratio	40	6.8	2.75	0.76	6.2	4.9–7.5	6.9	2.85	0.55	6.5	4.8–7.5	0.75	1.01	0.80–1.29	0.90
RV e’/a’ ratio	40	0.60	0.19	0.05	0.60	0.53–0.74	0.68	0.27	0.05	0.63	0.55–0.76	0.71	1.16	0.86–1.56	0.32
TV E velocity (cm/s)	40	31	4.4	1.2	30	29–33	34	7.8	1.5	36	29–41	0.14	2.27	0.79–6.52	0.13
TV E velocity (s^−^^1^)—normalised	40	12	1.8	0.5	12	11.2–12.9	16	3.3	0.63	16	14–18	0.001	1.60	1.15–2.23	0.006
TV A velocity (cm/s)	40	58	9.0	2.5	57	54–65	65	10.8	2.1	67	58–73	0.035	1.97	0.97–4.00	0.06
TV A velocity (s^−^^1^)—normalised	40	23	5.3	1.5	24	21–24	30	5.1	0.98	29	27–34	<0.001	1.32	1.08–1.62	0.006
TV E/A ratio	40	0.54	0.10	0.03	0.56	0.47–0.59	0.54	0.11	0.02	0.55	0.46–0.62	0.82	0.94	0.49–1.81	0.86
***RV Global Function***															
RV IVRT (ms)	40	38	10.4	2.87	37	33–41	39	14.6	2.81	37	28–49	1.00	1.01	0.96–1.06	0.70
RV IVCT (ms)	40	47	14.4	4.01	47	38–55	42	16.7	3.21	36	28–55	0.31	0.98	0.94–1.02	0.33
***Pulmonary Vascular Status***															
RV MPI (PW)	39	0.15	0.10	0.03	0.13	0.09–0.19	0.31	0.18	0.04	0.29	0.16–0.40	0.009	2.28	1.15–4.54	0.02
RV MPI (TDI)	40	0.56	0.13	0.04	0.59	0.44–0.66	0.56	0.21	0.04	0.53	0.44–0.61	0.59	1.00	0.70–1.42	0.99
PAAT (ms)	39	76	19	5.61	73	65–87	60	22	4.32	55	47–64	0.02	0.72	0.51–1.00	0.047
RVET (ms)	39	183	14	4.09	185	175–191	169	14	2.75	170	162–176	0.007	0.49	0.27–0.89	0.02
PAAT/RVET ratio	39	0.41	0.10	0.03	0.40	0.36–0.49	0.35	0.12	0.02	0.32	0.28–0.42	0.09	0.61	0.33–1.15	0.13

MWU = Mann–Whitney U test; LR = Logistic Regression; *p* = *p*-value; OR = Odds Ratio; CI = Confidence Interval; SD = Standard Deviation; SE = Standard Error; IQR = Interquartile Range. BPD = bronchopulmonary dysplasia; FAC = fractional area change; MAP = mean airway pressure; RV = right ventricle; s’ = systolic velocity; e’ = early diastolic velocity; a’ = late diastolic velocity; TAPSE = tricuspid annular plane systolic excursion; TV = tricuspid valve; IVRT = isovolumic relaxation time; IVCT = isovolumic contraction time; MPI = myocardial performance index; Norm = normalised; PW = pulsed wave; TDI = tissue Doppler imaging; PAAT = pulmonary artery acceleration time; RVET = right ventricular ejection time.

**Table 2:** Descriptive statistics for candidate variables at Scan 2 (Day 9). Comparison between No BPD and BPD groups.

Parameter	N	No BPD (n = 13)					BPD (n = 26)					MWU *p*	OR	95% CI	LR *p*
		Mean	SD	SE	Median	IQR	Mean	SD	SE	Median	IQR				
***Clinical Variables***															
Gestational Age (weeks)	40	29	2.31	0.64	30	28–31	26	1.88	0.36	26	25–27	<0.001	0.50	0.33–0.76	<0.001
Current Weight (g)	39	1302	353	98	1243	1095–1596	823	229	45	831	624–938	<0.001	0.54	0.37–0.81	0.003
MAP (cmH_2_O)	39	1.5	3.13	0.87	0.0	0.0–0.0	7.7	3.16	0.62	7.9	5.1–9.9	<0.001	1.74	1.26–2.40	<0.001
***RV Systolic Function***															
RV s’ (cm/s)—absolute	39	5.2	0.87	0.24	5.0	4.7–5.9	5.5	0.68	0.13	5.5	5.3–6.0	0.26	1.65	0.65–4.22	0.29
RV s’ (s^−^^1^)—normalised	39	2.0	0.47	0.13	1.9	1.7–2.3	2.4	0.41	0.08	2.4	2.1–2.7	0.01	2.76	1.11–6.89	0.03
TAPSE (cm)—absolute	39	0.68	0.11	0.03	0.68	0.61–0.77	0.64	0.13	0.02	0.63	0.57–0.67	0.3	0.78	0.45–1.38	0.39
TAPSE—normalised	39	0.26	0.05	0.01	0.26	0.24–0.29	0.28	0.05	0.01	0.28	0.26–0.31	0.3	1.95	0.46–8.27	0.36
RV FAC (%)	36	23	9.9	2.87	26	18–30	21	8.37	1.71	20	15–29	0.43	0.98	0.90–1.06	0.55
***RV Diastolic Function***															
RV e’ (cm/s)—absolute	39	6.5	1.09	0.30	6.7	5.7–7.0	5.7	1.09	0.21	5.8	4.9–6.2	0.048	0.50	0.24–1.02	0.06
RV e’ (s^−^^1^)—normalised	39	2.5	0.57	0.16	2.3	2.2–2.7	2.5	0.52	0.10	2.4	2.0–2.8	0.96	0.91	0.49–1.71	0.77
RV a’ (cm/s)—absolute	39	8.3	1.57	0.43	7.9	7.2–9.4	9.4	1.92	0.38	8.8	8.4–10.4	0.14	1.43	0.92–2.22	0.11
RV a’ (s^−^^1^)—normalised	39	3.3	0.81	0.22	3.1	2.7–3.5	4.1	0.85	0.17	4.1	3.2–4.4	0.009	1.93	1.13–3.28	0.02
RV E/e’ ratio	39	5.5	0.81	0.22	5.3	5.0–5.9	7.2	2.83	0.55	6.8	5.3–8.4	0.03	2.03	1.06–3.88	0.03
RV e’/a’ ratio	39	0.79	0.14	0.04	0.83	0.72–0.87	0.64	0.18	0.04	0.64	0.56–0.74	0.005	0.54	0.32–0.90	0.02
TV E velocity (cm/s)	39	35	6.8	1.88	37	31–38	40	12.1	2.37	39	32–43	0.30	1.62	0.73–3.60	0.24
TV E velocity (s^−^^1^)—normalised	39	14	2.88	0.8	14	12–14	17	5.35	1.05	16	14–19	0.01	1.26	1.00–1.59	0.046
TV A velocity (cm/s)	39	60	15	4.19	65	47–70	70	13	2.58	68	62–79	0.14	1.66	0.96–2.85	0.07
TV A velocity (s^−^^1^)—normalised	39	23	6.46	1.79	23	19–24	30	6.2	1.2	28	27–35	0.002	1.23	1.05–1.45	0.01
TV E/A ratio	39	0.60	0.09	0.02	0.61	0.51–0.66	0.58	0.16	0.03	0.56	0.50–0.65	0.52	0.90	0.55–1.46	0.66
***RV Global Function***															
RV IVRT (ms)	39	38	7.5	2.07	38	32–42	37	16.2	3.18	37	25–45	0.45	0.99	0.95–1.04	0.76
RV IVCT (ms)	39	35	13.5	3.75	31	24–44	30	12	2.36	27	23–36	0.21	0.97	0.92–1.02	0.22
***Pulmonary Vascular Status***															
RV MPI (PW)	38	0.21	0.19	0.05	0.16	0.09–0.23	0.34	0.27	0.05	0.29	0.17–0.47	0.08	1.37	0.91–2.08	0.14
RV MPI (TDI)	39	0.44	0.12	0.03	0.43	0.33–0.52	0.41	0.12	0.02	0.39	0.31–0.53	0.54	0.80	0.45–1.41	0.44
PAAT (ms)	38	76	17.5	5.06	75	67–82	69	24.0	4.77	65	47–85	0.31	0.87	0.64–1.19	0.39
RVET (ms)	38	183	19	5.58	190	177–194	167	19	3.79	166	158–184	0.009	0.61	0.38–0.97	0.04
PAAT/RVET ratio	38	0.42	0.11	0.03	0.40	0.38–0.45	0.41	0.13	0.03	0.41	0.32–0.50	0.84	0.94	0.54–1.64	0.83

MWU = Mann–Whitney U test; LR = Logistic Regression; *p* = *p*-value; OR = Odds Ratio; CI = Confidence Interval; SD = Standard Deviation; SE = Standard Error; IQR = Interquartile Range. BPD = bronchopulmonary dysplasia; FAC = fractional area change; MAP = mean airway pressure; RV = right ventricle; s’ = systolic velocity; e’ = early diastolic velocity; a’ = late diastolic velocity; TAPSE = tricuspid annular plane systolic excursion; TV = tricuspid valve; IVRT = isovolumic relaxation time; IVCT = isovolumic contraction time; MPI = myocardial performance index; Norm = normalised; PW = pulsed wave; TDI = tissue Doppler imaging; PAAT = pulmonary artery acceleration time; RVET = right ventricular ejection time.

**Table 3:** Descriptive statistics, comparison between No BPD and BPD groups, and ROC analysis at Scan 1 for PDA-related variables.

Parameter	No BPD (n = 13) Median (IQR)	BPD (n = 27) Median (IQR)	MWU *p*	AUC (95% CI)	Cutoff	Sens/Spec
PDA diameter—absolute (cm)	0.13 (0.04–0.18)	0.21 (0.18–0.24)	<0.001	0.829 (0.69–0.97)	≥0.19	70/85%
PDA diameter—normalised (dimensionless)	0.06 (0.01–0.07)	0.09 (0.08–0.12)	<0.001	0.869 (0.76–0.98)	≥0.07	82/85%
LA:Ao ratio	0.97 (0.81–1.12)	0.99 (0.83–1.35)	0.54	0.561 (0.37–0.75)	≥1.44	26/92%
LPA diastolic velocity (m/s)	0.17 (0.15–0.19)	0.39 (0.29–0.51)	0.02	0.724 (0.54–0.91)	≥0.27	78/85%
PDA diastolic velocity (m/s)	0.16 (0.00–1.63)	0.81 (0.43–1.35)	0.33	0.598 (0.38–0.82)	≥0.26	93/54%
MV E/A ratio	0.81 (0.72–0.85)	0.79 (0.64–0.86)	0.75	0.467 (0.29–0.65)	≥0.91	19/100%

MWU = Mann–Whitney U test; *p* = *p*-value; ROC = Receiver Operating Characteristic; AUC = Area Under the Curve; CI = Confidence Interval; Sens = Sensitivity; Spec = Specificity; IQR = Interquartile Range; PDA = patent ductus arteriosus; LA = left atrium; Ao = aorta; LPA = left pulmonary artery; MV = mitral valve; E = early diastolic velocity; A = late diastolic velocity. Cutoff values were derived using the Youden index.

**Table 4:** Descriptive statistics, comparison between No BPD and BPD groups, and ROC analysis at Scan 2 for PDA-related variables.

Parameter	No BPD (n = 13) Median (IQR)	BPD (n = 26) Median (IQR)	MWU *p*	AUC (95% CI)	Cutoff	Sens/Spec
PDA diameter—absolute (cm)	0.15 (0.05–0.16)	0.21 (0.11–0.25)	0.049	0.70 (0.52–0.87)	≥0.20	54/92%
PDA diameter—normalised (dimensionless)	0.06 (0.02–0.07)	0.08 (0.05–0.11)	0.02	0.73 (0.57–0.90)	≥0.07	54/92%
LA:Ao ratio	1.00 (0.70–1.24)	1.24 (1.03–1.47)	0.03	0.72 (0.55–0.89)	≥1.27	42/92%
LPA diastolic velocity (m/s)	0.16 (0.11–0.28)	0.31 (0.11–0.48)	0.36	0.59 (0.41–0.77)	≥0.29	58/77%
PDA diastolic velocity (m/s)	0.16 (0.00–0.93)	0.40 (0.19–0.82)	0.49	0.57 (0.36–0.78)	≥0.14	84/46%
MV E/A ratio	0.81 (0.69–0.89)	0.74 (0.62–0.87)	0.36	0.41 (0.23–0.59)	≥1.13	8/100%

MWU = Mann–Whitney U test; *p* = *p*-value; ROC = Receiver Operating Characteristic; AUC = Area Under the Curve; CI = Confidence Interval; Sens = Sensitivity; Spec = Specificity; IQR = Interquartile Range; PDA = patent ductus arteriosus; LA = left atrium; Ao = aorta; LPA = left pulmonary artery; MV = mitral valve; E = early diastolic velocity; A = late diastolic velocity. Cutoff values were derived using the Youden index.

**Table 5:** Univariable logistic regression: Odds ratios with 95% confidence intervals for the association between PDA-related variables and BPD at Scan 1 and Scan 2.

Parameter	Scan 1 OR (95% CI)	*p*-Value	Scan 2 OR (95% CI)	*p*-Value
PDA diameter—absolute (cm)	1.21 (1.07–1.38)	0.003	1.09 (1.00–1.18)	0.06
PDA diameter—normalised (dimensionless)	1.70 (1.21–2.39)	0.002	1.29 (1.04–1.60)	0.02
LA:Ao ratio	1.07 (0.86–1.33)	0.53	1.31 (1.02–1.68)	0.04
LPA diastolic velocity (m/s)	1.63 (1.06–2.51)	0.03	1.22 (0.88–1.69)	0.23
PDA diastolic velocity (m/s)	1.05 (0.94–1.16)	0.42	1.02 (0.92–1.13)	0.68
MV E/A ratio	1.04 (0.75–1.45)	0.81	0.86 (0.61–1.22)	0.39

OR = Odds Ratio; CI = Confidence Interval; PDA = patent ductus arteriosus; LA = left atrium; Ao = aorta; LPA = left pulmonary artery; MV = mitral valve; E = early diastolic velocity; A = late diastolic velocity.

**Table 6:** Six variable groups proposed for validation in a BPD prediction model.

Group	Domain	Variables (Total 26)	Key Measures
1	Clinical variables (3)	Gestational Age, Birth Weight, Mean Airway Pressure	Baseline risk profile
2	RV systolic function (3)	s’ (normalised), TAPSE (normalised), RV FAC%	Longitudinal contractility
3	RV function in diastole (11)	RV e’ (absolute/normalised), RV a’ (absolute/normalised), RV E/e’, TV E (absolute/normalised), TV A (absolute/normalised), TV E/A, RV e’/a’	Relaxation and filling
4	RV global function (2)	IVRT, IVCT	Overall RV efficiency
5	Pulmonary vascular status (6)	PAAT, RVET, PAAT/RVET, RV-MPI-TDI, RV-MPI-PW, TR Pg *	Pulmonary haemodynamics
6	PDA assessment (1)	Proposed cPDA Severity Scoring System ^†^	Shunt significance

RV = right ventricle; s’ = systolic tissue Doppler velocity; TAPSE = tricuspid annular plane systolic excursion; FAC = fractional area change; e’ = early diastolic tissue Doppler velocity; a’ = late diastolic tissue Doppler velocity; E = early diastolic velocity; A = late diastolic velocity; TV = tricuspid valve; IVRT = isovolumic relaxation time; IVCT = isovolumic contraction time; PAAT = pulmonary artery acceleration time; RVET = right ventricular ejection time; MPI = myocardial performance index; TDI = tissue Doppler imaging; PW = pulsed wave; TR = tricuspid regurgitation; Pg = pressure gradient; PDA = patent ductus arteriosus; cPDA = cerebral perfusion-focused PDA severity scoring system (Conceptual proposal for future research) ^†^. * Prospective candidate; not analysed in this cohort.

**Figure A1:**
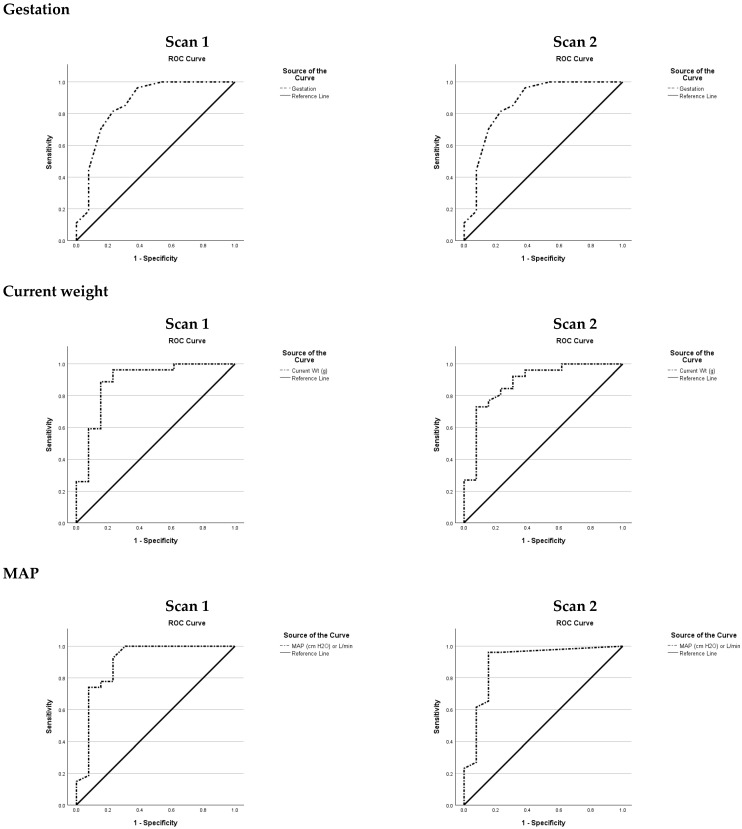
Clinical Variables.

**Figure A2:**
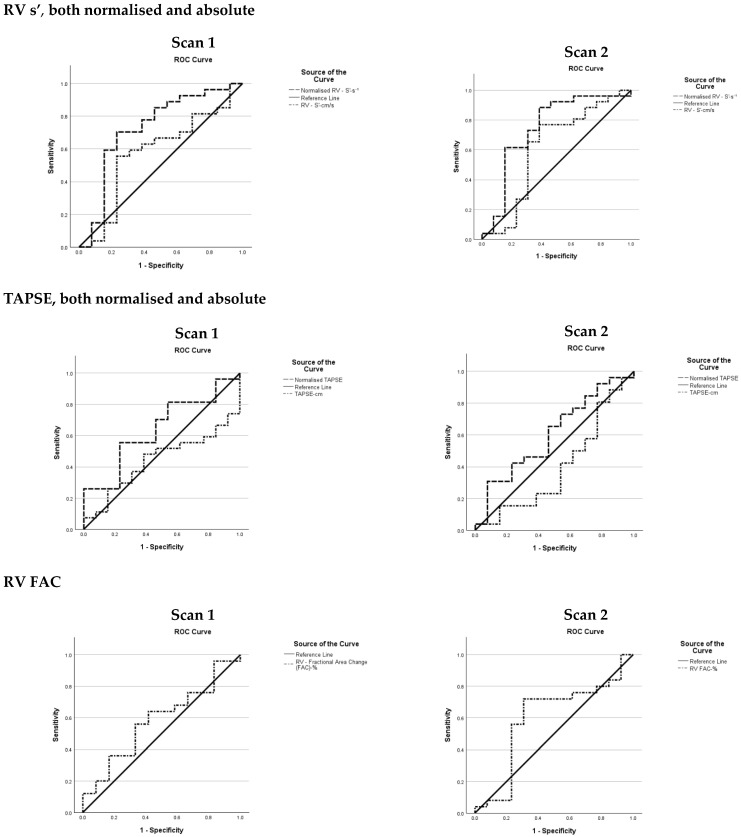
Right Ventricular (RV) Systolic Function Variables.

**Figure A3:**
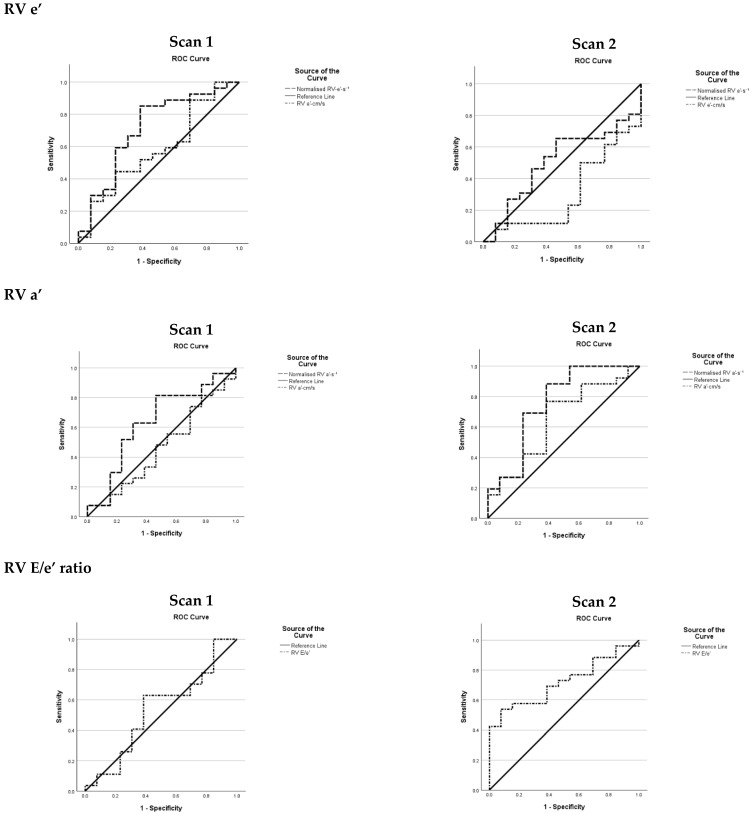

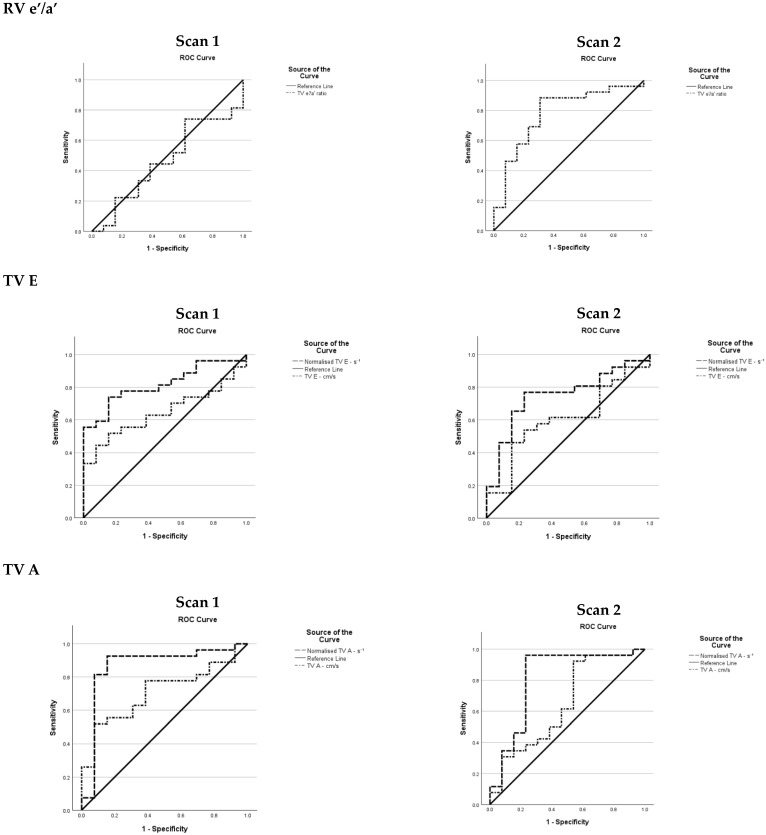

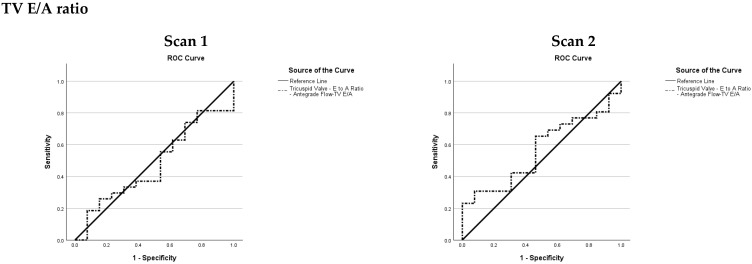
RV Function during Diastole Variables.

**Figure A4:**
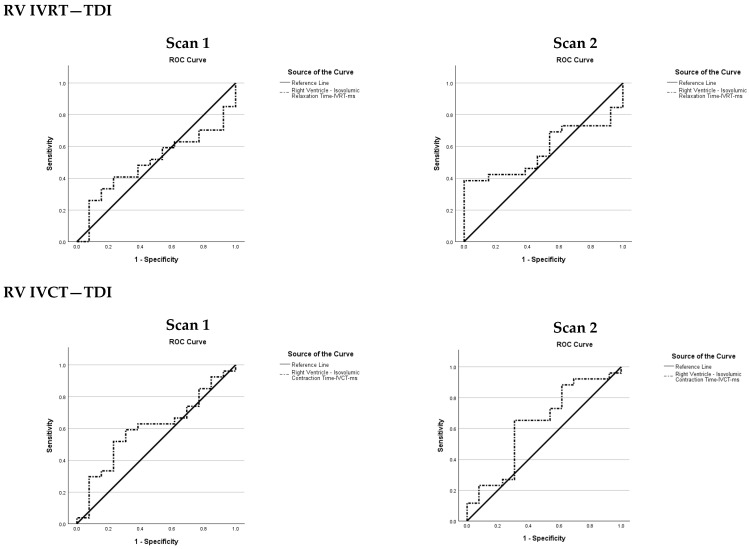
RV Global Function Variables.

**Figure A5:**
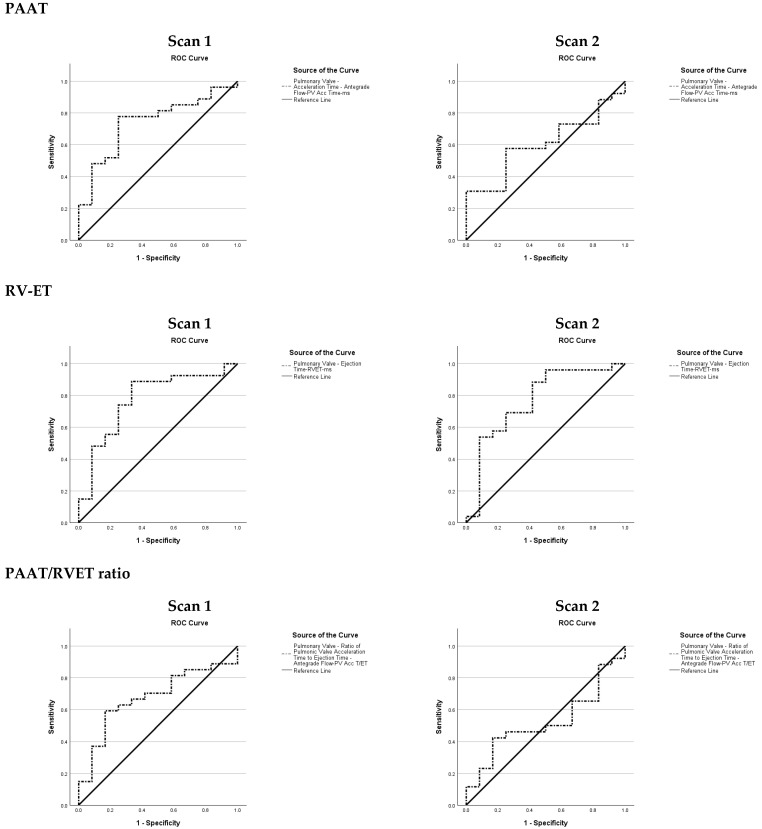

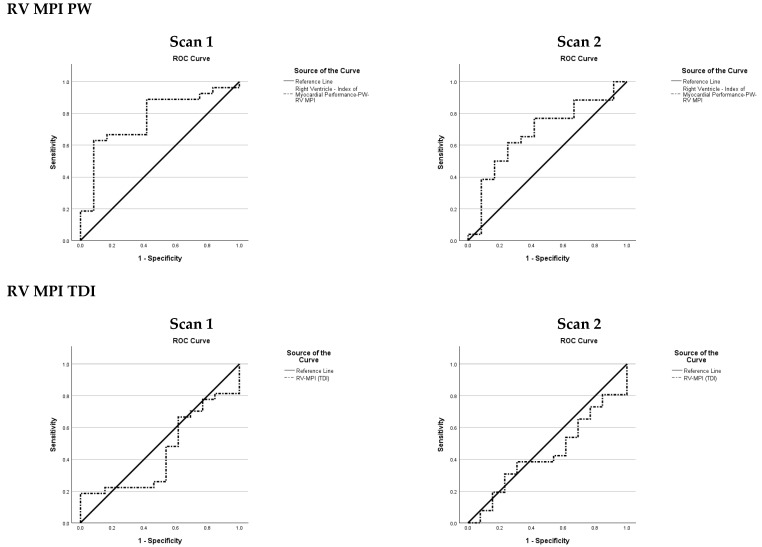
Pulmonary Vascular Status Variables.

**Figure A6:**
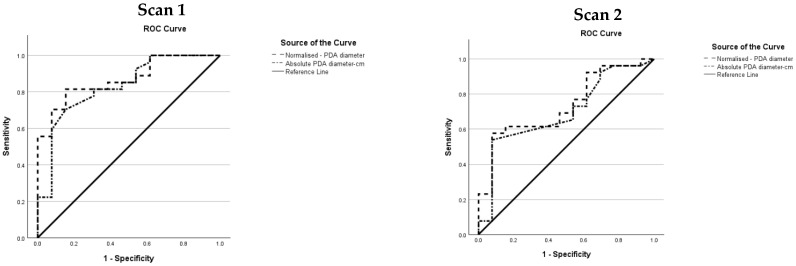
PDA diameter.

**Figure A7:**
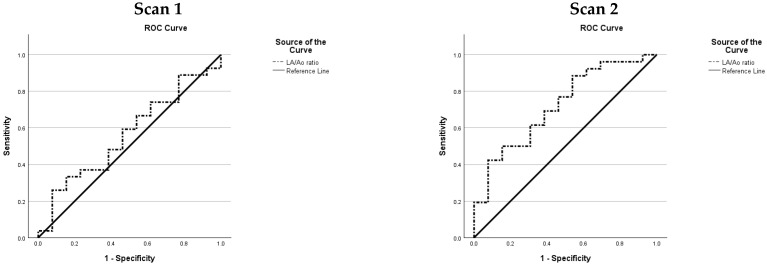
LA/Ao ratio.

**Figure A8:**
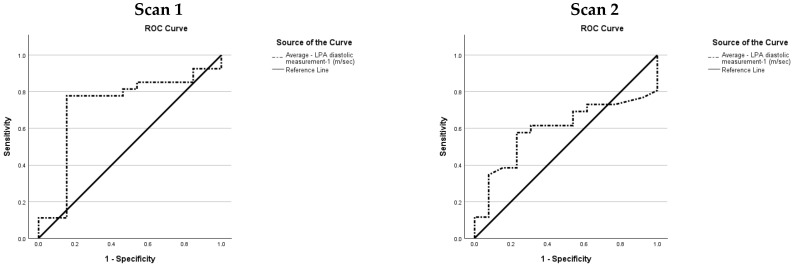
LPA diastolic velocity.

**Figure A9:**
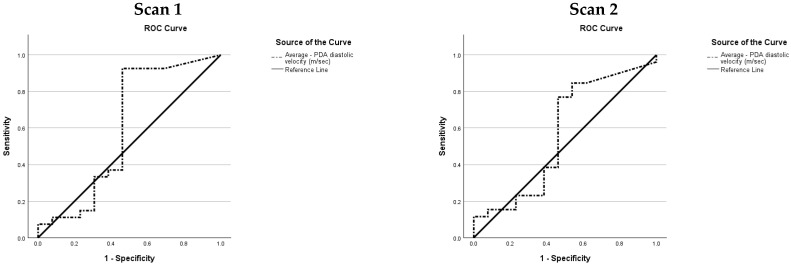
PDA diastolic velocity.

**Figure A10:**
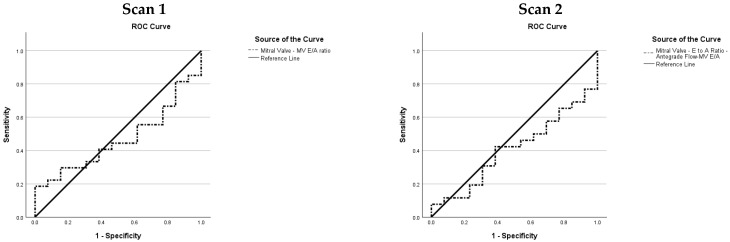
MV E/A ratio.

## Data Availability

The anonymised raw data supporting the conclusions of this article are not publicly available due to patient confidentiality and data protection policies of University Hospitals Plymouth NHS Trust. Data may be made available by the corresponding author on reasonable request and subject to approval from the study sponsor, University Hospitals Plymouth NHS Trust.

## Abbreviations

The following abbreviations are used in this manuscript:

2D CPA	Two-Dimensional Cardiac Performance Analysis
a’	Peak Late Diastolic Tissue Velocity
ACA	Anterior Cerebral Artery
AUC	Area Under the Curve
BPD	Bronchopulmonary Dysplasia
CI	Confidence Interval
cPDA	Cerebral Perfusion-Focused Patent Ductus Arteriosus Scoring System
DAB	Days After Birth
e’	Peak Early Diastolic Tissue Velocity
E/A	Early-to-Late Diastolic Velocity Ratio
E/e’	Early Diastolic Inflow to Early Diastolic Tissue Velocity Ratio
ECG	Electrocardiogram
FAC	Fractional Area Change
FiO_2_	Fraction of Inspired Oxygen
IQR	Interquartile Range
IRAS	Integrated Research Application System
IVCT	Isovolumic Contraction Time
IVRT	Isovolumic Relaxation Time
IVS	Interventricular Septum
LA:Ao	Left Atrium to Aortic Root Ratio
LR	Logistic Regression
LPA	Left Pulmonary Artery
MAP	Mean Airway Pressure
MPI	Myocardial Performance Index
MV	Mitral Valve
MWU	Mann–Whitney U test
NICU	Neonatal Intensive Care Unit
NPE	Neonatologist Performed Echocardiography
OR	Odds Ratio
PAAT	Pulmonary Artery Acceleration Time
PDA	Patent Ductus Arteriosus
PGmax	Peak Pressure Gradient
PH	Pulmonary Hypertension
PVR	Pulmonary Vascular Resistance
PW	Pulsed Wave
REPORT-BPD	Exploring Right vEntricular function applicability in a Prediction mOdel to identify pReterm infanTs with early Broncho-Pulmonary Dysplasia
ROC	Receiver Operating Characteristic
RV	Right Ventricle / Right Ventricular
RVET	Right Ventricular Ejection Time
s’	Peak Systolic Tissue Velocity
Sens	Sensitivity
Spec	Specificity
TAPSE	Tricuspid Annular Plane Systolic Excursion
TDI	Tissue Doppler Imaging
TR	Tricuspid Regurgitation
TV	Tricuspid Valve
Vmax	Maximum Velocity

## Appendix A

### Appendix A.2. Area Under the Curve (AUC) and Confidence Interval (CI) for Both Scans Clinical and Echo Indices Excluding PDA-Related Variables

**Variable**	**Scan 1**				**Scan 2**			
	**AUC**	**SE**	**95% CI**	***p***	**AUC**	**SE**	**95% CI**	***p***
**Gestation**	0.86	0.072	0.72–1.00	0.000	0.86	0.072	0.72–1.00	0.000
**Current weight**	0.89	0.064	0.76–1.01	0.000	0.88	0.062	0.76–1.00	0.000
**MAP**	0.89	0.065	0.77–1.03	0.000	0.89	0.064	0.77–1.02	0.000
**RV s’**	0.58	0.102	0.38–0.78	0.459	0.61	0.108	0.40–0.82	0.298
**Normalised RV s’**	0.73	0.094	0.54–0.91	0.017	0.75	0.094	0.56–0.93	0.009
**TAPSE**	0.44	0.090	0.26–0.62	0.520	0.39	0.101	0.20–0.60	0.307
**Normalised TAPSE**	0.64	0.091	0.46–0.82	0.123	0.60	0.097	0.41–0.79	0.286
**RV FAC**	0.61	0.092	0.43–0.79	0.229	0.60	0.105	0.40–0.80	0.336
**RV e’**	0.58	0.098	0.39–0.77	0.407	0.30	0.089	0.13–0.48	0.027
**RV a’**	0.46	0.099	0.27–0.65	0.719	0.65	0.096	0.46–0.84	0.123
**RV E/e’ ratio**	0.53	0.102	0.33–0.73	0.748	0.72	0.081	0.56–0.88	0.006
**RV e’/a’**	0.54	0.092	0.35–0.73	0.693	0.78	0.082	0.62–0.94	0.001
**TV E**	0.65	0.085	0.48–0.81	0.085	0.60	0.094	0.42–0.79	0.270
**TV A**	0.71	0.083	0.55–0.87	0.011	0.65	0.099	0.45–0.84	0.137
**TV E/A ratio**	0.48	0.096	0.29–0.66	0.801	0.57	0.094	0.382–0.75	0.487
**RV IVRT—TDI**	0.50	0.093	0.32–0.68	0.988	0.58	0.090	0.40–0.75	0.395
**RV IVCT**	0.60	0.094	0.42–0.79	0.282	0.63	0.097	0.43–0.82	0.198
**RV MPI PW**	0.77	0.082	0.61–0.93	0.001	0.68	0.094	0.50–0.86	0.057
**RV MPI TDI**	0.44	0.096	0.26–0.63	0.566	0.44	0.095	0.25–0.63	0.515
**PAAT**	0.74	0.084	0.58–0.91	0.004	0.61	0.092	0.43–0.79	0.252
**RV-ET**	0.78	0.085	0.61–0.94	0.001	0.77	0.090	0.59–0.95	0.003
**PAAT/RVET ratio**	0.68	0.089	0.50–0.85	0.049	0.52	0.097	0.33–0.71	0.816
SE = Standard Error; CI = Confidence Interval; AUC = Area Under the Curve.								
